# Consanguinity-based analysis of exome sequencing yields likely genetic causes in patients with inherited retinal dystrophy

**DOI:** 10.1186/s13023-021-01902-5

**Published:** 2021-06-15

**Authors:** Ren-Juan Shen, Jun-Gang Wang, Yang Li, Zi-Bing Jin

**Affiliations:** 1 Beijing Institute of Ophthalmology, Beijing Tongren Eye Center, Beijing Tongren Hospital, Capital Medical University, Beijing Ophthalmology & Visual Sciences Key Laboratory, Beijing, China; 2 Department of Ophthalmology, Eye Hospital of Shandong First Medical University, State Key Laboratory Cultivation Base, Shandong Provincial Key Laboratory of Ophthalmology, Shandong Eye Institute, Shandong First Medical University & Shandong Academy of Medical Sciences, Jinan, China

**Keywords:** Inherited retinal dystrophy, Consanguinity, Exome sequencing, Genetic mutation

## Abstract

**Background:**

Consanguineous families have a relatively high prevalence of genetic disorders caused by bi-allelic mutations in recessive genes. This study aims to evaluate the effectiveness and efficiency of a consanguinity-based exome sequencing approach to capturing genetic mutations in inherited retinal dystrophy families with consanguineous marriages.

**Methods:**

Ten unrelated consanguineous families with a proband affected by inherited retinal dystrophy were recruited in this study. All participants underwent comprehensive ophthalmic examinations. Whole exome sequencing was performed, followed by a homozygote-prior strategy to rapidly filter disease-causing mutations. Bioinformatic prediction of pathogenicity, Sanger sequencing and co-segregation analysis were carried out for further validation.

**Results:**

In ten consanguineous families, a total of 10 homozygous mutations in 8 IRD genes were identified, including 2 novel mutations, c.1654_1655delAG (p. R552Afs*5) in gene *FAM161A* in a patient diagnosed with retinitis pigmentosa, and c.830T > C (p.L277P) in gene *CEP78* in a patient diagnosed with cone and rod dystrophy.

**Conclusion:**

The genetic etiology in consanguineous families with IRD were successfully identified using consanguinity-based analysis of exome sequencing data, suggesting that this approach could provide complementary insights into genetic diagnoses in consanguineous families with variant genetic disorders.

**Supplementary Information:**

The online version contains supplementary material available at 10.1186/s13023-021-01902-5.

## Background

Inherited retinal dystrophy (IRD) is a group of severe and irreversible vision-threatening disorders, affecting approximately 2 million people worldwide [1]. It is characterized by degeneration and dysfunction of specialized and light-sensitive neurons in the retina, such as cone and/or rod photoreceptor cells [2–4]. IRD is comprised of a wide spectrum of diseases with enormous genetic heterogeneity [1, 5–7]. The most common form of IRD is retinitis pigmentosa (RP), which is already known to be attributed to the primary loss of rods, followed by secondary loss of cones. RP initially affects night and peripheral vision, but eventually, patients will experience central vision impairment [8, 9]. Other diseases in this group include cone and rod dystrophy (CORD), Leber congenital amaurosis (LCA), Bietti crystalline dystrophy (BCD) and syndromic diseases such as Usher syndrome and Bardet-Biedl syndrome [10–13]. Currently, more than 250 disease-causing genes have been identified in patients with IRD. Transmission of these genes has been shown to resemble Mendelian traits, including autosomal recessive (AR), autosomal dominant (AD), and X-linked (XL) modes of inheritance. Although great efforts have been made to dissect the genetic basis of IRD, the genetic causes of IRD in a large proportion of patients remain unknown [14–16].

Many communities around the world have a long tradition of marrying to relatives. The highest prevalence of inbreeding was observed in North and sub-Saharan Africa, the Middle East, and West, Central, and South Asia [17]. In the rural areas of China, consanguineous marriages such as cousin couples are relatively common, especially among minority ethnic groups. According to data from a population-based survey in the 1990s, the average consanguinity marriage rate among Chinese Han population was 1.4% and it was significantly higher in rural areas than in urban areas, while among Yi population which is one of the major ethnic minority of China, the rate was as high as 14.6% [18]. In the recent decade, nationwide quantitative information on consanguinity is not available, but some population surveys conducted in ethnic minority settlements indicate that the situation of consanguineous marriage has not changed much.

Consanguinity increases the likelihood of pathogenic mutations in a homoallelic state. A higher prevalence of rare hereditary diseases caused by homozygous mutations in AR genes has been observed among members of consanguineous families compared to those of non-consanguineous families [19, 20]. Several studies have also reported genetic causes of IRDs among consanguineous families worldwide, especially in the areas where consanguinity rate is particularly high [21–24].

However, few studies were performed among patients with IRD in Chinese consanguineous families. Our in-house data show that about 11% of IRD probands have consanguineous parents. Therefore, there is a need for genetic screening and counseling among with IRD patients in Chinese consanguineous families.

To date, next-generation sequencing (NGS) techniques have been widely applied to the research of IRDs, among which whole exome sequencing (WES) has demonstrated the greatest reliability and efficiency in testing genetic mutations [25–27]. In this study, deep sequencing of all causative genes of IRD was performed on ten probands, and WES data were analyzed with a consanguinity-based analysis strategy. This study not only broadened the genetic mutation spectrum but also enriched knowledge on the genotype–phenotype correlations of IRDs, suggesting that molecular genetic testing using a consanguinity-based approach is of great value for the diagnosis of IRDs.

## Methods

### Subjects and clinical evaluations

This study was conducted in accordance with the Declaration of Helsinki and was approved by the Ethics Committee of Beijing Tongren Hospital. Informed consent was acquired from all participating subjects. Ten probands, together with one affected sibling and 23 unaffected family members were recruited from ten unrelated consanguineous families. Medical history was obtained and comprehensive ophthalmic examinations, including fundus photography, optical coherence tomography (OCT), visual field testing, and electroretinogram (ERG), were performed. Peripheral blood samples from all participants were collected.

### Exome sequencing and homozygote strategy

Exome sequencing and subsequent procedures, including quality control and variation annotation, were performed as previously described [28–31]. Homozygous mutant alleles in all 281 known IRD genes were filtered first. Known IRD genes were gathered based on RetNet [32] (https://sph.uth.edu/RETNET/), OMIM (https://www.omim.org/) and numerous scientific literatures. After the implementation of the homozygote-prior filtering strategy, the remaining variants were confirmed by Sanger sequencing. Co-segregation analysis was performed in all available subjects from the ten families registered in this study.

### Assessment of pathogenicity

Variants with a minor allele frequency (MAF) of > 0.5% for recessive genes and > 0.01% for dominant genes in the 1000 Genomes Project (1000G), Exome Aggregation Consortium (ExAC) and Genome Aggregation Database (gnomAD) were excluded. The pathogenicity of each candidate variant was predicted by online analysis programs. Missense variants were evaluated with Mutation Taster (http://www.mutationtaster.org/), PolyPhen-2 (http://genetics.bwh.harvard.edu/pph2/), SIFT (http://provean.jcvi.org/protein) and CADD (https://cadd.gs.washington.edu/). Predicted crystal structures of the wild-type proteins and mutants were obtained using SWISS-MODEL (http://swissmodel.expasy.org) and demonstrated by PyMol software. Finally, the pathogenicity of variants was categorized as pathogenic, likely-pathogenic, uncertain significance, benign and likely benign according to ACMG (American College of Medical Genetics and Genomics) classification guidelines [33].

## Results

### Clinical features

In the current study, six probands were diagnosed with RP, while the other four probands were non-RP and their diagnoses included CORD, BCD, and LCA (Table 1).

**Table 1:** Clinical features of 10 probands

ID	Sex	Diagnosis	Initial sympton	NYS	Age of onset (y)	Age at visit (y)	BCVA (decimal) OD/OS	ERG (scotopic/photopic)	VF
F1-III:4	M	RP, HM	Night blindness	N	39	46	0.4/0.6	NA	Constricted
F2-III:3	M	RP, MC	Night blindness. Central vision impairment	N	< 1	27	0.1/0.1	Both severely reduced	NA
F3-III:5	F	RP	Night blindness	N	20	51	LP	NA	NA
F4-III:4	F	RP	Night blindness	N	20	60	HM/HM	Both severely reduced	NA
F5-III:1	M	RP	Night blindness	N	14	34	0.5/0.6	Both severely reduced	Constricted
F6-III:1	F	RP	Night blindness	N	6	23	0.8/0.6	Severely reduced/slightly reduced	Constricted
F7-III:4	M	CORD, POAG	Central vision impairment	N	40	45	CF at 1 m/0.02	Both severely reduced	Central scotoma
F8-III:5	F	CORD	Central vision impairment	N	32	43	CF at 40 cm/0.01	Both severely reduced, greater loss in cone	Central scotoma
F9-III:2	M	BCD	Night blindness	N	43	44	0.6/0.8	Severely reduced/slightly reduced	Constricted
F10-III:1	F	LCA	Vision impairment	Y	< 1	29	HM/HM	Extinguished	NA

NYS, Nystagmus; M, male; F, female; RP, retinitis pigmentosa; HM, high myopia; MC, coloboma-like lesions in macula; CORD, cone and rod dystrophy; POAG, primary open-angle glaucoma; BCD, Bietti crystalline dystrophy; BCVA, best corrected visual acuity; OD, right eye; OS, left eye; VF, visual field; ERGs, Electroretinograms; NA, not available

**Fig. 1:**
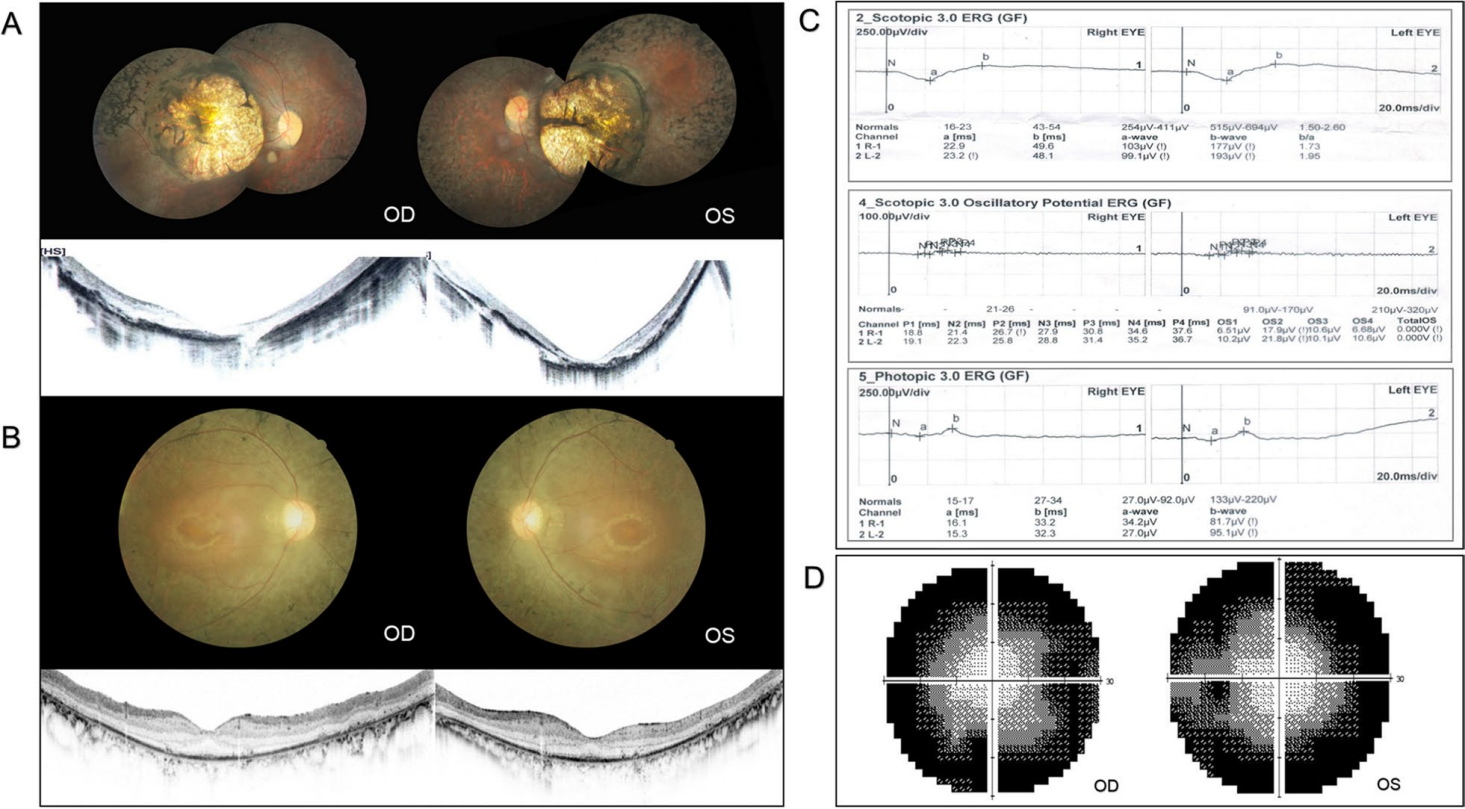
Clinical manifestations of typical and atypical RP patients. **A** Fundus photos of F2-III:3 presented bilateral prominent macular atrophy, with chorioretinal attenuation and extensive bone spicule pigmentation. OCT showed severe thinning of macular structure along with loss of photoreceptors. **B–D** Clinical manifestations of F5-III:1. **B** Fundus photos showed typical RP presentations, including waxy pallor disc, attenuated retinal vasculature, and mid-peripheral bone-spicule pigmentation. OCT illustrated severe loss of photoreceptors with preservation in the fovea. **C** Full-field electroretinogram (ERG) demonstrated a reduced rod and cone response amplitude. **D** Constricted visual field

Among the six RP probands, four presented as classic RP (F3-III:5, F4-III:4, F5-III:1 and F6-III:1). They complained of night blindness as the initial symptom, with progressive constriction of visual field presenting in the following years. Typical RP manifestations were observed in proband F5-III:1 (Fig. 1A). Atypical RP was observed in proband F1-III:4 and F2-III:3. F1-III:4 was diagnosed with RP and high myopia, and manifested typical RP fundus combined with a large myopic conus and a tigroid fundus change. F2-III:3 had atypical RP with involvement of the macula. Fundus photography demonstrated bilateral prominent coloboma-like lesions in the macula. OCT showed a severely thinned macula structure and lack of full-thickness in the inner and outer segments of photoreceptors (Fig. 1).

**Fig. 2:**
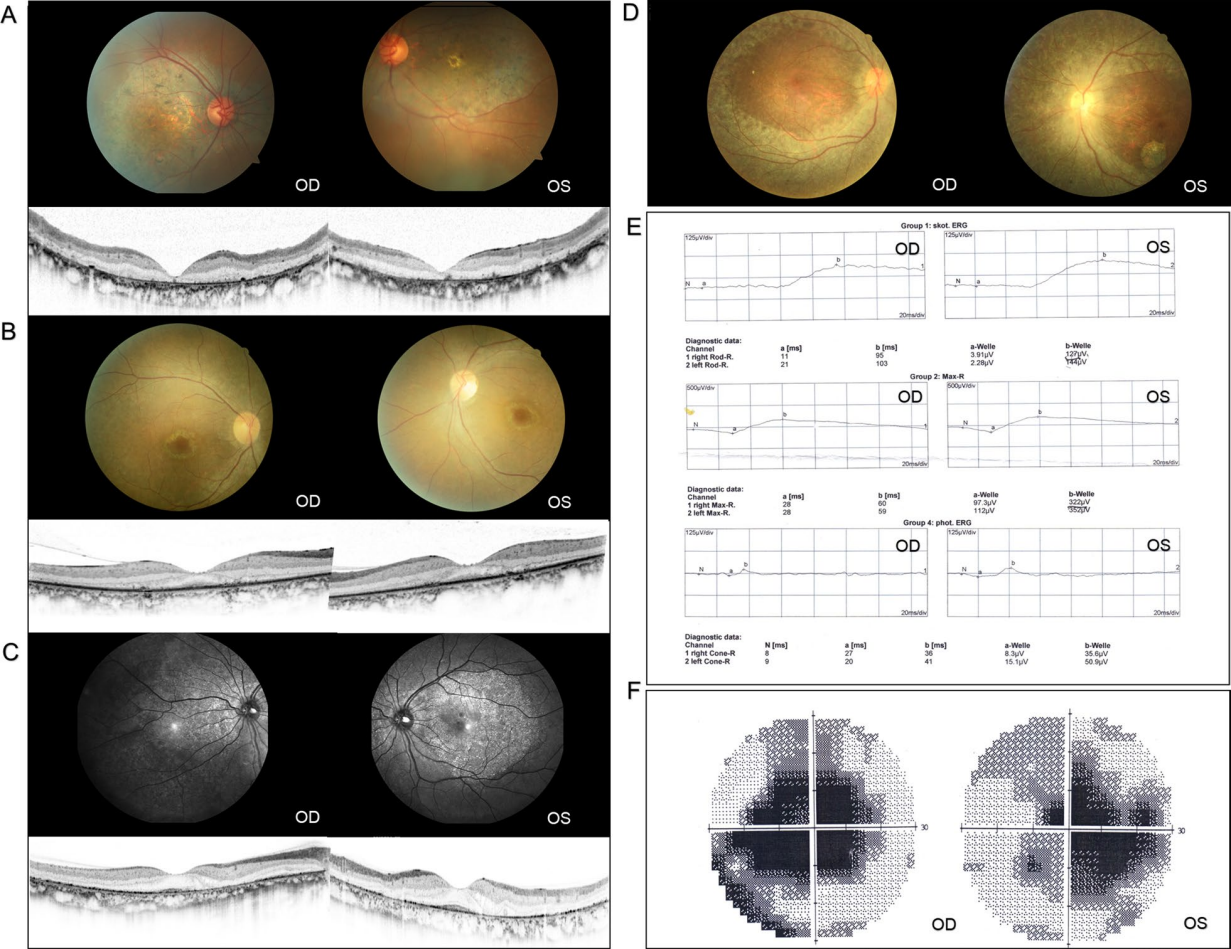
Clinical manifestations of non-RP patients. **A** Fundus photos of F7-III:4 diagnosed with CORD revealed an enlargement of the optic cup with a C/D of 0.4, decrease of neuroretinal rim width in the inferior and superior, attenuated retinal vasculature, and retinal pigment epithelium (RPE) changes over the macular region. OCT showed the absence of foveal photoreceptors and RPE. **B** Fundus and OCT of F8-III:5 diagnosed with CORD presented Bull’s eye maculopathy with loss of foveal photoreceptor and a blurred outer layer structure. **C** Fundus and OCT images of F9-III:2 diagnosed with BCD showed bilateral multiple crystalline deposits and RPE dystrophy with loss of photoreceptors. **D** Fundus images of F10-III:1 diagnosed with LCA exhibited show a waxy pallor optic disc, attenuated retinal vasculature, and extensive chorioretinal atrophy. **E** Full-field ERG of F7-III:4 manifested a significantly decreased amplitude of cones while the response of rods declined slightly in both eyes. **F** Visual field test of F7-III:4 revealed a serious impairment of the central visual field

Out of four probands with non-RP, F7-III:4 and F8-III:5 were diagnosed with CORD. They suffered a decrease of central vision in their 3rd or 4th decade, and BCVA had declined to 0.02 and 0.01 (decimal) respectively at their visit time. F7-III:4 was diagnosed with CORD combined with primary open-angle glaucoma (POAG). Fundus photography revealed an enlargement of the optic cup with a cup to disc ratio (C/D) as 0.4, a decrease of neuroretinal rim width in the inferior and superior, and a distinct appearance of maculopathy, including pigment degeneration and deposition over the macular region. OCT showed the absence of foveal photoreceptors and retinal pigment epithelium (RPE). In addition, the visual field test revealed a serious impairment of the central visual field. The full-field ERG showed that the amplitude of cones became evidently flat while that of rods declined slightly (Fig. 2A, D, E). HRT F8-III:5 was diagnosed with CORD and presented loss of foveal photoreceptor with a blurred outer layer structure (Fig. 2B), and ERG showed a greater loss of cone responses compared with rod responses.

F9-III:2 and F10-III:1 were diagnosed as BCD and LCA respectively, and typical disease manifestations such as multiple crystalline deposits in BCD and extensive chorioretinal atrophy in LCA were observed (Fig. 2).

### Molecular diagnosis

A gene list comprised of 281 known IRD-causing genes was screened and evaluated for implicated mutations by whole exome sequencing, with an overall average coverage of over 150 X (Additional file 1: Table S1).

**Fig. 3:**
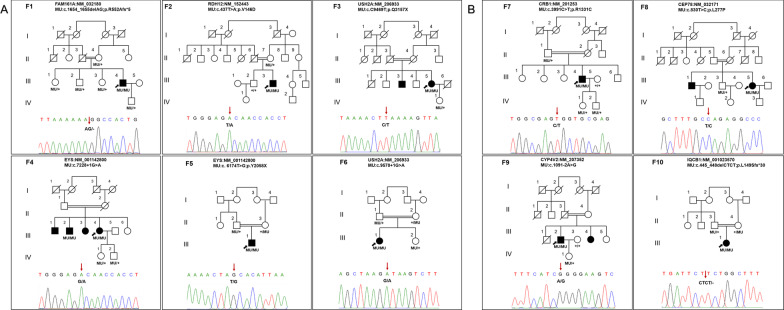
Pedigrees and segregation data. Pedigrees and segregation data of families diagnosed with RP and non-RP are illustrated in **A** and **B**, respectively. Squares and circles represent males and females. Filled symbols represent the affected patients. Arrows indicate the probands. MU: mutation. + : wildtype

Through this homozygote-prior strategy, the disease-causing mutations in all 10 families were identified in a highly efficient manner, with a total of 10 homozygous mutations in 8 IRD genes determined, including 8 previously reported mutations, and 2 novel mutations involving genes *FAM161A* in patient F1-III:4 with RP and *CEP78* in F8-III:5 with CORD (Table 2, Fig. 3). The identified mutations segregated with phenotype within all pedigrees.

**Table 2:** Genetic mutations identified in this study

ID	Gene	Mutation	Protein change	Transcript	MuationTaster	Poly Phen-2	Sift	CADD	gnomAD	References
F1-III:4	*FAM161A*	c.1654_1655delAG	p.R552Afs*5	NM_032180.3	D	/	/	/	0	Novel
F2-III:3	*RDH12*	c.437 T > A	p.V146D	NM_152443.3	D	D	D	27	0.00001193	Gong et al. [34]
F3-III:5	*USH2A*	c.9469C > T	p.Q3157X	NM_206933.4	D	/	/	37	0.00001631	Jiang et al. [35]
F4-III:4	*EYS*	c.7228 + 1G > A	splicing	NM_001142800.2	D	/	/	25.9	0.00002783	Gu et al. [36]
F5-III:1	*EYS*	c.6174 T > G	p.Y2058X	NM_001142800.2	D	/	/	37	0	ClinVar submitted
F6-III:1	*USH2A*	c.9570 + 1G > A	splicing	NM_206933.4	D	/	/	24.6	0.0000408	Xu et al. [37]
F7-III:4	*CRB1*	c.3991C > T	p.R1331C	NM_201253.3	D	D	D	32	0.00001989	Tsang et al. [38]
F8-III:5	*CEP78*	c.830 T > C	p.L277P	NM_032171.3	P	D	D	23.2	0.00003244	Novel
F9-III:2	*CYP4V2*	c.1091-2A > G	splicing	NM_207352.4	D	/	/	33	0.00004771	Yin et al. [39]
F10-III:1	*IQCB1*	c.445_448delCTCT	p.L149Sfs*30	NM_001023570.4	D	/	/	/	0	Otto et al. [40]

Homo, homozygous; Mutation Taster-D, disease causing; P, polymorphism; Polyphen-2-D, probably damaging; Sift-D, damaging

The deletion of nucleotides in *FAM161*, c.1654_1655delAG (p. R552Afs*5) resulted in frameshift mutations and affected the normal RNA maturation process, leading to a production of truncated protein. This frameshift mutation had a severe impact on protein function and was absent in the gnomAD database, and therefore, by definition in ACMG guidelines, was a pathogenic mutation.

**Fig. 4:**
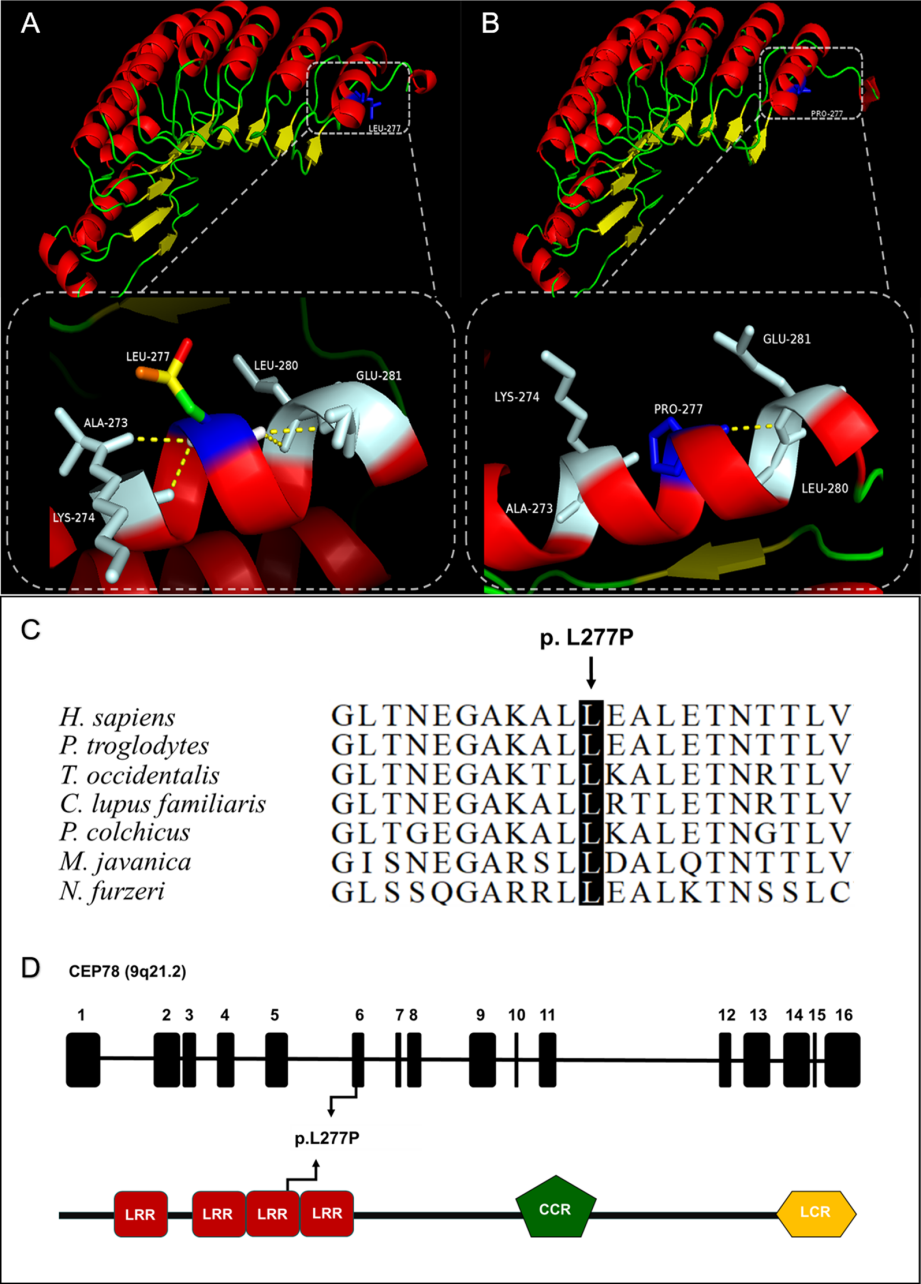
Evaluation of L277P mutation in CEP78. **A** Presentation of wildtype CEP78 protein 3D structure. **B** Mutant status of CEP78 protein. The amino acid at position 277 was mutated from leucine (LEU) to proline acid (PRO), leading to the loss of three hydrogen bonds between the wildtype LEU at residue 277 and Alanine acid (ALA) at residue 273, Lysine acid (LYS) at residue 274, and LEU at residue 280. **C** Alignment of CEP78 protein sequence from amino acid 267 to 287 to its orthologous protein sequences in different species indicated evolutionary conservation of leucine at position 277 in human CEP78. **D** Representations of relative linear locations of the L277P mutation in genome structure (top) and protein domains (bottom)

F8-III:5 was found to harbor a homozygous novel missense mutation, c.830T>C (p.L277P) in *CEP78*. Leucine acid (LEU) at residue 277 is located in a region of a highly conserved domain called leucine-rich repeat (LRR), which presents in a number of proteins with diverse functions. Although Mutation Taster defines the mutation as polymorphism, Sift, Poly-Phen 2, Mutation Assessor and CADD all define it as pathogenic. And the variant had an extremely low allele frequency in healthy control cohort (Table 2). The three-dimensional protein crystal model of *CEP78* was constructed to verify the structure impact of p.L277P with PyMOL. As the model showed, the substitution of LEU for proline acid (PRO) resulted in the loss of three hydrogen bonds between the wildtype LEU at residue 277 and Alanine acid (ALA) at residue 273, Lysine acid (LYS) at residue 274, and LEU at residue 280 (Fig. 4). Considering the above, c.830T>C (p.L277P) in *CEP78* is categorized as likely pathogenic according to the ACMG guideline (PM2+PM3+PP3+PP4).

## Discussion

Consanguineous marriage is prevalent in rural China, which could potentially increase the risk of suffering from hereditary diseases. In consanguineous families, genetic defects that are caused by homozygous mutations inherited respectively from both parents who are carriers of the heterozygous mutation often manifest in the offspring. Incidence of IRDs, for example, could lead to devastating visual disability and negative livelihood impact. However, few studies focused on Chinese consanguineous families with IRDs. To broaden the knowledge on this population, deep exome sequencing and consanguinity-based analysis was performed in order to elucidate the genetic etiology in patients with IRD.

In this study, 10 homozygous disease-causing mutations were successfully identified. As for the genetic pathogenesis of four typical RP families, *USH2A* and *EYS* mutations accounted for 50%, which was consistent with our previous study showing that *USH2A* and *EYS* are among the most common disease-causing genes of ARRP in Chinese population [14].

F1-III:4 was diagnosed with RP and high myopia and was identified to harbor a novel homozygous mutation c.1654_1655delAG (p. R552Afs*5) within *FAM161A*. To date, *FAM161A* mutations have only been linked to ARRP, and mostly are in the form of null mutations [41]. A previous study did observe that RP patients associated with *FAM161A* mutation manifested moderate-high myopia, which was consistent with our observation [42]. The protein encoded by *FAM161A* is evolutionarily conserved in vertebrates such as Pan troglodytes, Macaca mulatta, Equus asinus and Sarcophilus harrisii. It localizes to photoreceptor connecting cilia in human and ciliary basal body in mammalian ciliated cells. Recent research has demonstrated that this protein is involved in microtubule acetylation and stabilization by maintaining transport processes in photoreceptors as a component of the cilia-basal body complex [43, 44]. *FAM161A* directly interacts with cilia proteins *CEP290*, *SDCCAG8*, *OFD1* and lebercilin, all of which are implicated in heterogeneous IRDs [45].

*CEP78* has been reported to be related to the pathogenesis of Usher syndrome or cone dystrophy along with sensory hearing loss in recent studies [46, 47]. Patient F8-III:5 in our study had a homozygous missense mutation c.830T > C (p.L277P) in *CEP78* and presented cone and rod dystrophy without hearing impairment. The mutation is very likely to be the genetic cause based on the evidences above, but the phenotype is unusual as all patients reported to harbor *CEP78* mutations have hearing impairment. Our report of the clinical manifestation indicates that *CEP78*-related IRDs may have a high clinical heterogeneity. However, further research and clinical observation are needed.

A frameshift mutation c.445-448delCTCT (p. Leu149Serfs*31) in gene *IQCB1* was determined in F10-III:1, a patient who was diagnosed with LCA. This mutation was pathogenic according to ACMG guidelines, and has been reported in two unrelated German families to be related to Senior-Loken syndrome [40]. Senior-Loken syndrome is a rare autosomal recessive ciliopathy characterized by the association of nephronophthisis (NPHP) and early onset retinal dystrophy. Age of onset, severity and progression of symptoms can vary greatly among affected individuals. Patient F10-III:1 suffered from severe vision impairment in the first year of her life and no kidney disorder was found in the following 30 years. This may be a novel correlation between *IQCB1* mutation and complex clinical manifestations. However, regular follow-up at nephrology department is needed.

F2-III:3 was characterized by a rare type of RP associated with macular and choroid coloboma, and had a homozygous mutation c.437T > A (p.V146D) in *RDH12*. *RDH12* coded protein is predominantly expressed in the inner segment of rod and cone photoreceptors as an NADPH-dependent reductase involved in the conversion of all-trans-retinal and 11-cis-retinal to the corresponding retinols [48]. This p.V146D mutation was previously reported in severe early-onset ARRP family [34]. In contrast, the proband in this current study exhibited additional phenotypes. A prior study also indicated that *RDH12* mutation could lead to LCA with macular coloboma [49]. However, in our study, characteristic features of LCA, including nystagmus, photophobia, amaurotic pupils and flat or nondetectable ERG, were all absent [50].

F7-III:4 was diagnosed with CORD and harbored a homozygous mutation c.3991C > T (p.R1331C) in *CRB1*. CRB1 localizes to the outer limiting membrane of the retina and is expressed in adherens junction in Muller glia cells, which may control retinogenesis via signaling pathway such as mTOR, Hippo, Wnt and Notch1 [51].The p.R1331C mutation was reported to have caused unusual maculopathy in an Irish family [38]. In contrast, F7-III:4 had a definite diagnosis of CORD with a wider range of fundus atrophy and evident ERG performance. Furthermore, the patient suffered from more severe binocular glaucoma and cataract. Our findings have verified the implication of the RP or LCA-related *CRB1* mutation in maculopathy in a Chinese individual.

**Fig. 5:**
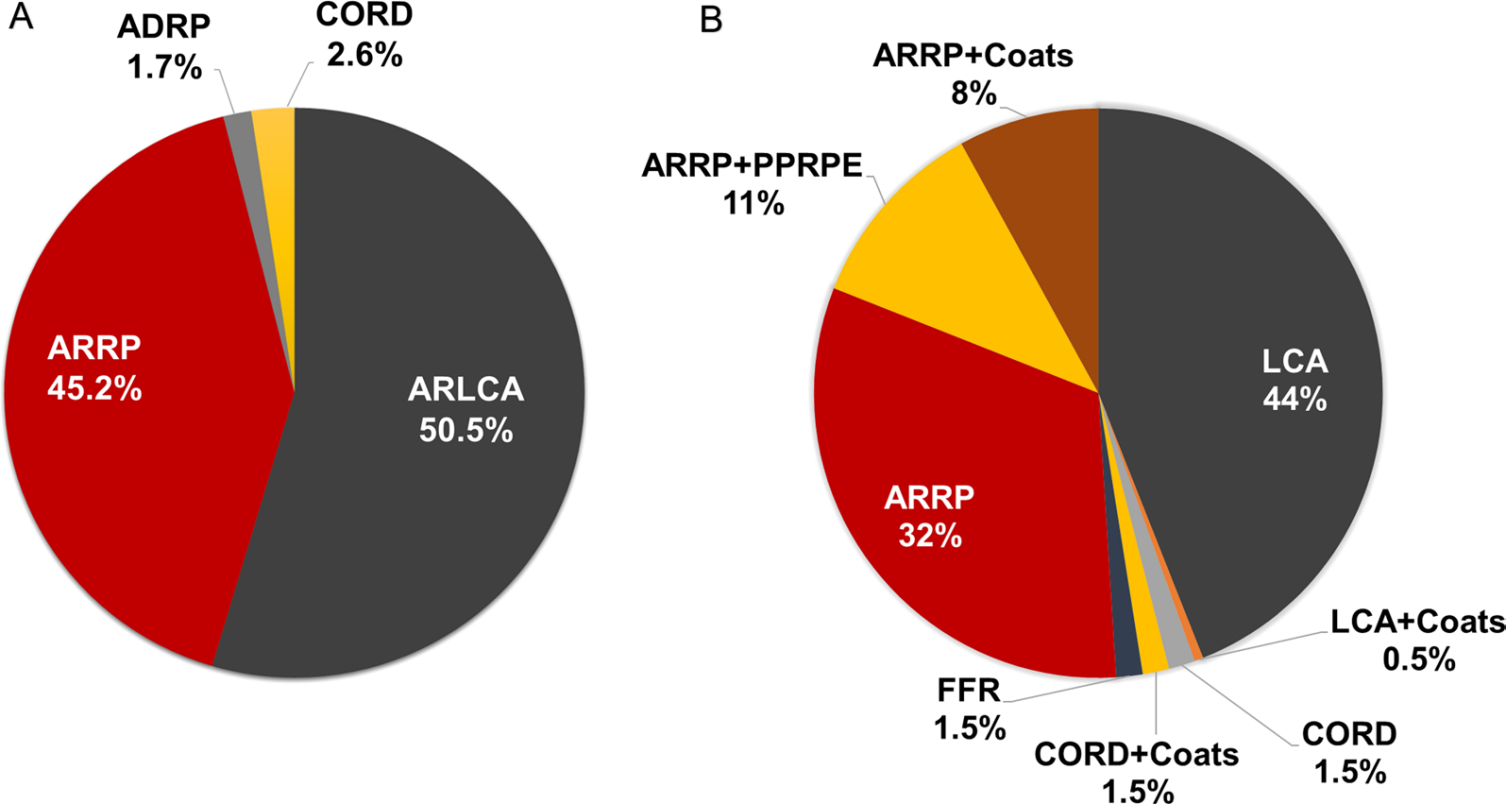
Proportion of correlated phenotypes of mutated *RDH12* and *CRB1.* Pie charts illustrate the proportion of heterogeneous phenotypes correlated with *RDH12* mutations (**A**) and *CRB1* mutations (**B**). ARRP: autosomal recessive retinitis pigmentosa. ADRP: autosomal dominant retinitis pigmentosa. ARLCA: autosomal recessive Leber congenital amaurosis. CORD: cone and rod dystrophy. PPRPE: preserved para-arteriole retinal pigment epithelium. FFR: familial foveal retinoschisis

To date, more than 110 mutations within *RDH12* have been classified to be responsible for either RP, LCA or CORD. And more than 150 *CRB1* mutations are shown to be associated with specific fundus features other than RP or LCA, such as preserved para-arteriole retinal pigment epithelium (PPRPE) and retinal telangiectasia with exudation (Coats-like exudative vasculopathy) [52, 53]. Moreover, some mutations within *CRB1* were also causative of Familial foveal Retinoschisis (FFR) [54]. We reviewed *RDH12* and *CRB1*-related disorders from public databases such as ClinVar (https://www.ncbi.nlm.nih.gov/clinvar/) and HGMD (http://www.hgmd.cf.ac.uk), and summarized the proportion of heterogeneous phenotypes correlated with *RDH12* and *CRB1* mutations in Fig. 5. Some researches indicate that additional modifying factors could be responsible for the absence of clear correlation between mutations and phenotypes. For example, a previous study has demonstrated an earlier onset and more severe retinal phenotype when *Mthfr* and rd8 mutations coexist in mice, indicating that Mthfr is a modify factors of *Crb1* [55].

The strategy we use in this study would help achieving quick and accurate localization of the possible disease-causing mutations, thus identifying the genetic cause of diseases efficiently and cost-effectively. However, there are some limitations. For example, focusing only on homozygous variations may lead to missing of de novo mutations, as well as the underlying disease-causing mutation in an AD or X linked pattern of hereditary. And due to the extreme genetic and clinical heterogeneity of IRD, as well as the existence of de novo mutation, copy number variation and structural variation, this approach is not 100% successful. In order to solve the genetic causes of patients with negative results, further mining is needed. Homozygosity mapping or detection of runs of homozygosity (ROH) through WES data can be performed to detect novel candidate genes using several practical and reliable tools. AutoMap, for example, was conducted and tested to be one of the best homozygosity mapping tools by a similar recent study in Iranian families. With AutoMap, the researchers predicted ROHs from WES data with high specificity and sensitivity, and also obtain a high detection rate in their IRD cohort [56]. De novo mutations should arouse great attention and whole genome sequencing (WGS) and long-read sequencing can play reliable roles in detecting large structural variations.

## Conclusion

Our study employed a consanguinity-based exome sequencing analysis approach to dissect the genetic causes of IRDs in ten consanguineous Chinese families. We revealed two novel mutations and genotype–phenotype correlations, thereby expanding our understanding of the underlying heterogeneous mechanism of IRDs. Moreover, this current study attested to the value of molecular genetic testing using a consanguinity-based approach in the diagnosis of recessive genetic defects, providing a perspective for determining novel pathogenic genes in a more cost-effective and efficient way.

## Supplementary Information

**Additional file 1: Table S1**. List of IRD genes.

## Data Availability

The datasets used and/or analyzed during the current study are available from the corresponding author on reasonable request.

## Abbreviations

IRD: Inherited retinal dystrophy
RP: Retinitis pigmentosa
CORD: Cone and rod dystrophy
LCA: Leber congenital amaurosis
BCD: Bietti crystalline dystrophy
AR: Autosomal recessive
AD: Autosomal dominant
XL: X-linked
NGS: Next-generation sequencing
WES: Whole exome sequencing
OCT: Optical coherence tomography
ERG: Electroretinogram
POAG: Primary open-angle glaucoma
RPE: Retinal pigment epithelium
ACMG: American College of Medical Genetics and Genomics
LEU: Leucine acid
LRR: Leucine-rich repeat
PRO: Proline acid
ALA: Alanine acid
LYS: Lysine acid
PPRPE: Para-arteriole retinal pigment epithelium
FFR: Familial foveal retinoschisis
