# Supplementary material for: Consanguinity-based analysis of exome sequencing yields likely genetic causes in patients with inherited retinal dystrophy
Source: Orphanet J Rare Dis. 2021 Jun 15;16:278. doi: 10.1186/s13023-021-01902-5 (PMC8204521; doi:10.1186/s13023-021-01902-5)
Supplement: Supplementary file 1 — Additional file 1: Table S1. List of IRD genes. [file 13023_2021_1902_MOESM1_ESM.docx]

**Supplementary Table 1. List of IRD genes.**

| Genes associated with IRD | | | | | | | | | | | |
| --- | --- | --- | --- | --- | --- | --- | --- | --- | --- | --- | --- |
| ABCA4 | ABCC6 | ABHD12 | ACBD5 | ACO2 | ADAM9 | ADAMTS18 | ADGRV1 | ADIPOR1 | AFG3L2 | AGBL5 | AHI1 |
| AHR | AIPL1 | ALMS1 | ARHGEF18 | ARL2BP | ARL3 | ARL6 | ARMS2 | ARSG | ASRGL1 | ATF6 | ATXN7 |
| BBIP1 | BBS1 | BBS10 | BBS12 | BBS2 | BBS4 | BBS5 | BBS7 | BBS9 | BEST1 | C12orf65 | C1QTNF5 |
| C2 | C21orf2 | C2orf71 | C3 | C8orf37 | CA4 | CABP4 | CACNA1F | CACNA2D4 | CAPN5 | CC2D2A | CCT2 |
| CDH23 | CDH3 | CDHR1 | CEP164 | CEP19 | CEP250 | CEP290 | CEP78 | CERKL | CFB | CFH | CHM |
| CIB2 | CLCC1 | CLN3 | CLRN1 | CLUAP1 | CNGA1 | CNGA3 | CNGB1 | CNGB3 | CNNM4 | COL11A1 | COL2A1 |
| COL9A1 | CRB1 | CRX | CSPP1 | CTNNA1 | CYP4V2 | DFNB31 | DHDDS | DHX38 | DMD | DRAM2 | DTHD1 |
| EFEMP1 | ELOVL1 | ELOVL4 | EMC1 | ERCC6 | ESPN | EXOSC2 | EYS | FAM161A | FBLN5 | FLVCR1 | FSCN2 |
| FZD4 | GDF6 | GNAT1 | GNAT2 | GNB3 | GNPTG | GPR125 | GPR179 | GRK1 | GRM6 | GUCA1A | GUCA1B |
| GUCY2D | HARS | HGSNAT | HK1 | HMCN1 | HMX1 | HTRA1 | IDH3B | IFT140 | IFT172 | IFT27 | IFT81 |
| IMPDH1 | IMPG1 | IMPG2 | INPP5E | INVS | IQCB1 | ITM2B | JAG1 | KCNJ13 | KCNV2 | KIAA1549 | KIF11 |
| KIZ | KLHL7 | KSS | LAMA1 | LCA5 | LHON | LRAT | LRIT3 | LRP5 | LZTFL1 | MAK | MAPKAPK3 |
| MERTK | MFN2 | MFRP | MFSD8 | MIR204 | MKKS | MKS1 | MT-ATP6 | MT-TH | MT-TL1 | MTTP | MT-TP |
| MT-TS2 | MVK | MYO7A | NBAS | NDP | NEK2 | NEUROD1 | NMNAT1 | NPHP1 | NPHP3 | NPHP4 | NR2E3 |
| NR2F1 | NRL | NYX | OAT | OFD1 | OPA1 | OPA3 | OPN1LW | OPN1MW | OPN1SW | OTX2 | PANK2 |
| PAX2 | PCDH15 | PCYT1A | PDE6A | PDE6B | PDE6C | PDE6G | PDE6H | PDZD7 | PEX1 | PEX7 | PGK1 |
| PHYH | PITPNM3 | PLA2G5 | PLK4 | PNPLA6 | POC1B | POC5 | POMGNT1 | PRCD | PRDM13 | PROM1 | PRPF3 |
| PRPF31 | PRPF4 | PRPF6 | PRPF8 | PRPH2 | PRPS1 | RAB28 | RAX2 | RB1 | RBP3 | RBP4 | RCBTB1 |
| RD3 | RDH11 | RDH12 | RDH5 | REEP6 | RGR | RGS9 | RGS9BP | RHO | RIMS1 | RLBP1 | ROM1 |
| RP1 | RP1L1 | RP2 | RP9 | RPE65 | RPGR | RPGRIP1 | RPGRIP1L | RS1 | RTN4IP1 | SAG | SAMD11 |
| SDCCAG8 | SEMA4A | SLC24A1 | SLC25A46 | SLC7A14 | SNRNP200 | SPATA7 | SPP2 | TEAD1 | TIMM8A | TIMP3 | TLR3 |
| TLR4 | TMEM126A | TMEM216 | TMEM237 | TOPORS | TREX1 | TRIM32 | TRNT1 | TRPM1 | TSPAN12 | TTC8 | TTLL5 |
| TTPA | TUB | TUBGCP4 | TUBGCP6 | TULP1 | UNC119 | USH1C | USH1G | USH2A | VCAN | VSX2 | WDPCP |
| WDR19 | WFS1 | ZNF408 | ZNF423 | ZNF513 |  |  |  |  |  |  |  |
